# Exercise-Induced Elevated BDNF Level Does Not Prevent Cognitive Impairment Due to Acute Exposure to Moderate Hypoxia in Well-Trained Athletes

**DOI:** 10.3390/ijms21155569

**Published:** 2020-08-04

**Authors:** Zofia Piotrowicz, Małgorzata Chalimoniuk, Kamila Płoszczyca, Miłosz Czuba, Józef Langfort

**Affiliations:** 1 Institute of Sport Sciences, The Jerzy Kukuczka Academy of Physical Education, 40-065 Katowice, Poland; langfort@imdik.pan.pl; 2 Department of Tourism and Health in Biała Podlaska, The Józef Piłsudski University of Physical Education, 00-968 Warsaw, Poland; malgorzata.chalimoniuk@awf-bp.edu.pl; 3 Department of Kinesiology, Institute of Sport, 01-982 Warsaw, Poland; kamila.ploszczyca@insp.waw.pl (K.P.); milosz.czuba@insp.waw.pl (M.C.); 4 Faculty of Health Sciences, Jan Dlugosz University, 42-200 Czestochowa, Poland

**Keywords:** brain-derived neurotrophic factor, moderate hypoxia, physical exercise, psychomotor function, reaction time, cortisol, catecholamines, nitrite, endotheline-1, lactate

## Abstract

Exposure to acute hypoxia causes a detrimental effect on the brain which is also manifested by a decrease in the ability to perform psychomotor tasks. Conversely, brain-derived neurotrophic factor (BDNF), whose levels are elevated in response to exercise, is a well-known factor in improving cognitive function. Therefore, the aim of our study was to investigate whether the exercise under hypoxic conditions affects psychomotor performance. For this purpose, 11 healthy young athletes performed a graded cycloergometer exercise test to volitional exhaustion under normoxia and acute mild hypoxia (FiO_2_ = 14.7%). Before, immediately after exercise and after a period of recovery, choice reaction time (CRT) and number of correct reactions (NCR) in relation to changes in serum BDNF were examined. Additionally, other selected factors which may modify BDNF production, i.e., cortisol (C), nitrite, catecholamines (adrenalin-A, noradrenaline-NA, dopamine-DA, serotonin-5-HT) and endothelin-1 (ET-1), were also measured. Exercise in hypoxic conditions extended CRT by 13.8% (*p* < 0.01) and decreased NCR (by 11.5%) compared to rest (*p* < 0.05). During maximal workload, NCR was lower by 9% in hypoxia compared to normoxia (*p* < 0.05). BDNF increased immediately after exercise in normoxia (by 29.3%; *p* < 0.01), as well as in hypoxia (by 50.0%; *p* < 0.001). There were no differences in BDNF between normoxia and hypoxia. Considering the fact that similar levels of BDNF were seen in both conditions but cognitive performance was suppressed in hypoxia, acute elevation of BDNF did not compensate for hypoxia-induced cognition impairment. Moreover, neither potentially negative effects of C nor positive effects of A, DA and NO on the brain were observed in our study.

## 1. Introduction

Hypoxia is a condition in which some organ(s) or a whole organism is deprived of adequate oxygen supply. Except in very short or static exercises [1,2,3], hypoxia negatively affects exercise performance [4,5]. In particular, the maximal aerobic workload that can be sustained during exercise involving large muscle groups (e.g., cycling, running) is considerably lower in hypoxia compared with normoxia. The origin of human performance limitation in hypoxia is attributed to a decrease in maximal oxygen uptake (VO_2max_). Dempsey and Wagner [6] observed that each 1% decrement in SaO_2_% below the 95% level approximates to a 1–2% decrement in VO_2max_. Diminished VO_2max_ in hypoxia is accompanied by a lowered O_2_ partial pressure in arterial blood (PaO_2_), which reduces O_2_ delivery to tissues and negatively affects muscle metabolism and contraction [7,8], leading to so-called peripheral fatigue. There is also evidence that maximal cardiac output and maximal heart rate (HR_max_) during maximal exercise in hypoxia are decreased [9] and the decrease in HR_max_ is linearly related to the decrease in SaO_2_% [10]. This effect can be reversed by oxygen administration during hypoxia exposure in both acute [11] and chronic hypoxia [9,11,12,13].

The aforementioned factors do not fully explain the hypoxia-induced reduction in exercise performance. As biochemical, electromyographic and mechanical signs of muscle fatigue are reduced in severe hypoxia compared with normoxia, peripheral (muscle) fatigue may not be the main factor responsible for impaired exercise performance [12,14]. It is well recognized that metabolites produced in working muscles can directly modulate central nervous system (CNS) functions by changes in sensory nerve impulses. Moreover, chemical messengers originating from working muscle are released into the circulation [15] and can affect brain function after their translocation to the CNS. An alternative hypothesis that may, at least partially, explain reduced exercise performance in hypoxic condition attributes it to so-called central fatigue [16]. This assumption is supported by studies showing impairment of cognitive performance [17] by reduced O_2_ delivery. Several studies [18,19] have reported that moderate levels of hypoxia degraded the ability to perform psychomotor tasks, and the main cause of cognitive impairment is the low PaO_2_ regardless of the type of hypoxia (normobaric vs. hypobaric) [20].

The negative impact of hypoxia on cognitive functions is manifested by memory deterioration, reduced learning ability, decreased concentration, and psychomotor performance [21]. One of the best indicators of the speed and efficiency of mental processes is choice reaction time (CRT) and the number of correct reactions (NCR), especially if these variables are used to assess the cognitive function within the same group of participants [22,23]. However, in previous studies, the effect of hypoxia on CRT was ambiguous and most likely depended on the time of exposure [24,25] and level of hypoxia: moderate vs. severe [24,26,27]. Some studies show that acute exposure to severe hypoxia led to an increase in CRT [24,27]. However, prolonged exposure to moderate hypoxia did not disturb CRT [24,27]. Animal studies reveal that hypoxia causes neuronal injuries in the hippocampus and cortex, leading to functional and behavioral deficits [28,29,30]. Likewise, data obtained from neuroimaging proved that intermittent hypoxia may result in a decrease in the volume of the hippocampus in humans [31].

Some previous studies performed in normoxia indicated that exercise of low or moderate intensity improves psychomotor performance [32,33,34,35,36,37], while other studies showed a significant decrease in psychomotor performance during heavy exhaustive exercise [34,37,38]. It is not known if these aforementioned effects can be modulated by a hypoxia-induced deleterious influence on the CNS. Most recent data suggest that an essential role in these phenomena is played by brain-derived neurotrophic factor (BDNF) (for review, see [39,40]).

BDNF plays a key role in the physiology of the developing and mature CNS, showing a high affinity for the TrkB receptor. Consequently, it is responsible for neurogenesis, differentiation, survival, and remodeling of neurons, and it also positively affects synaptogenesis, synaptic plasticity, and long-term potentiation [41,42,43]. The upregulation of BDNF may influence brain functions including learning and memory [44].

Several lines of evidence suggest a link between BDNF and physical activity. Both acute and chronic aerobic activity were effective for increasing peripheral BDNF concentrations [45]. An elevated level of BDNF was also seen in active sportsman compared to sedentary individuals [46,47]. Another factor that can be considered a stimulator of BDNF production within the brain is nitric oxide (NO). A role for NO in increased BDNF production in response to exercise has been recently evidenced [48,49]. Exercises of extreme intensity or duration are known to greatly elevate blood cortisol (C) [50,51], while high circulating corticosterone has been shown to suppress brain production of BDNF in rats [52]. Of importance, BDNF induces expression of the monocarboxylate transporter that enables the use of lactate as an alternative energy source [53].

There are also very limited data about the efficacy of hypoxia exposure on psychomotor performance where subjects performed exercise with low and high intensity. Some data suggest that exposure to hypoxia reduces cognitive functions [17]. Also, mechanisms of hypoxia’s effect on the CNS are still poorly understood. One possible candidate which might take part in this phenomenon is BDNF. It has been shown that cognitive impairment is noticeable in neurodegenerative diseases, which is associated with a lower serum BDNF level as compared to healthy individuals [54]. Moreover, the level of this decrease depends on the degree of cognitive impairment [54]. On the other hand, BDNF is thought to be responsible for improving cognitive function as a result of exercise effort [55]. Moreover, it has been proven that both resting and post-exercise peripheral BDNF levels correspond to its brain production [45,56] and its level is elevated in response to exercise effort [46].

Therefore, the aim of this study was to examine the impact of a single bout of exercise to volitional exhaustion during acute exposure of well-trained endurance athletes to moderate hypoxia on psychomotor performance. For this purpose, we measured a peripheral level of BDNF and CRT and NCR as indices of psychomotor performance during graded cycloergometer exercise test. Furthermore, we examined a level of selected circulating biochemical factors, such as C, NO pathway-related metabolites (nitrite, endothelin-1(ET-1), catecholamines), because they are known to affect BDNF expression/production [48,49,52,57,58,59,60] and their expression can be influenced by both exercise and hypoxia [34,48,49,50,51,61,62,63,64,65,66,67,68].

## 2. Results

### 2.1. Maximal Workload and Respiratory Variables

**Figure 1:**
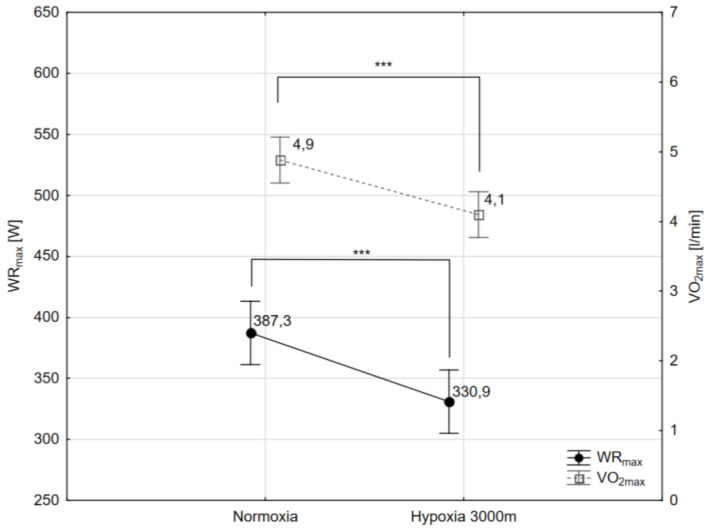
Maximal workload (WR_max_), and maximal oxygen uptake (VO_2max_) during incremental test performed in different conditions. *** *p* < 0.001.

**Figure 2:**
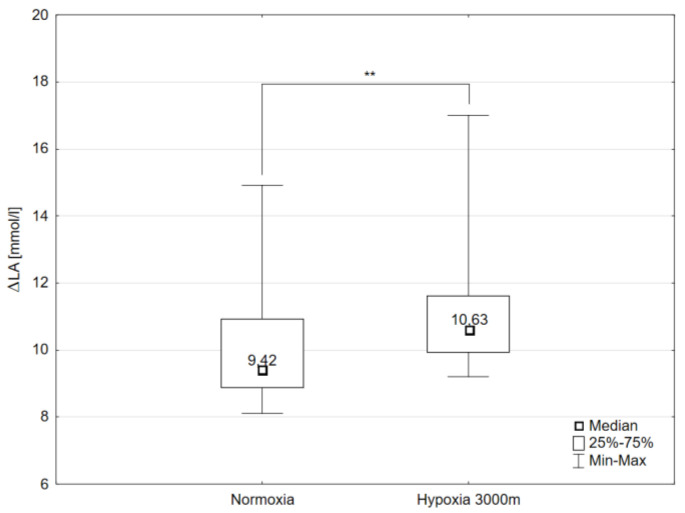
Delta values of blood lactate concentration (ΔLA) during incremental test performed in different conditions. ** *p* < 0.01.

The paired sample t-test showed that maximal workload (WR_max_) decreased significantly (*p* < 0.001) by 16.3% in hypoxia (3000 m) compared to the initial measurements in normoxia (Figure 1). The same trend of changes was observed in VO_2max_ values. The values of VO_2max_ decreased significantly (*p* < 0.001) in hypoxia compared to normoxia respectively by 14.5% (Figure 1). Additionally, there were statistically significant changes in delta values of blood lactate concentration (ΔLA) after the incremental test between normoxia and hypoxia 3000 m. The Wilcoxon test showed that ΔLA increased significantly (*p* < 0.01) by 6.05% despite the significant reduction in WR_max_ in hypoxia 3000 m compared to the measurements in normoxia (Figure 2).

### 2.2. Choice Reaction Time and Number of Correct Reactions

No significant interaction (condition x time of measure) effect was found on CRT but only a significant main effect of time of measurement (at rest, max and after 3 min of recovery) on CRT values (F = 21.88; *p* < 0.001) was observed. Additionally, there was a significant interaction (condition x time of measure) effect in the NCR (F = 3.44; *p* < 0.05) during the incremental test.

**Figure 3:**
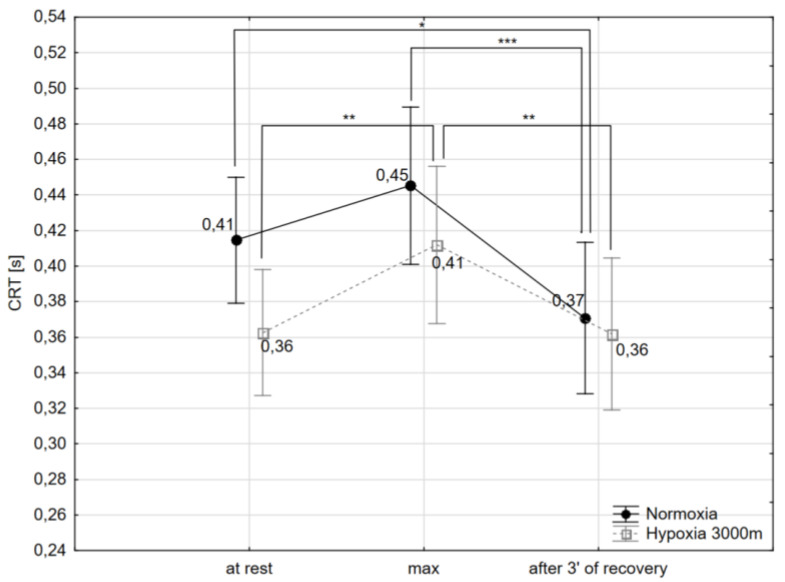
Choice reaction time (CRT) at rest, during maximal effort (max) and after 3 min of the recovery period in normoxia and hypoxia (3000 m). * *p* < 0.05; ** *p* < 0.01; *** *p* < 0.001.

The post-hoc Tukey’s test showed that CRT decreased significantly (*p* < 0.05) by 9.7% after 3 min of recovery after the incremental test (CRT_after 3′ of recovery_) compared to initial values observed at rest (CRT_rest_) in normoxia. Additionally, the values of CRT_after 3′ of recovery_ decreased significantly in normoxia (*p* < 0.001) and hypoxia (*p* < 0.01) compared to CRT during maximal workload of the incremental test (CRT_max_) respectively by 17.7 and 12.2% However, CRTmax increased significantly (*p* < 0.01) by 13.8% compared to CRT_rest_ in hypoxic conditions (Figure 3).

**Figure 4:**
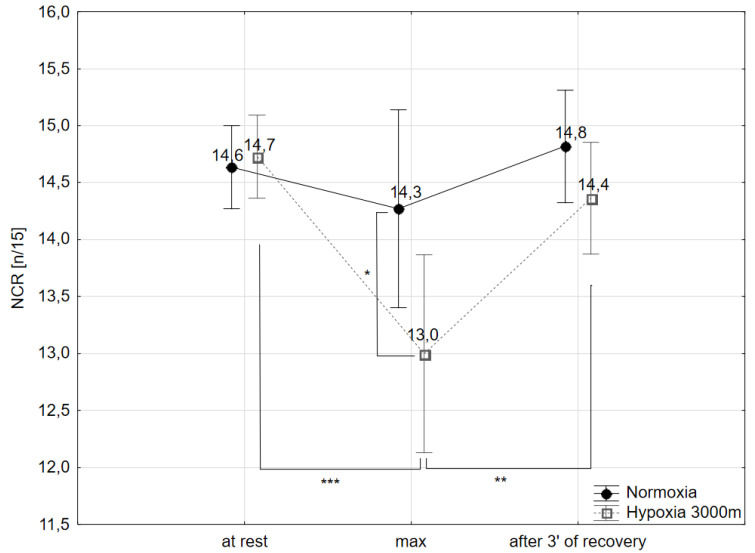
Number of correct reactions (NCR) at rest, during maximal effort (max) and after 3 min of the recovery period in normoxia and hypoxia (3000m). * *p* < 0.05; ** *p* < 0.01; *** *p* < 0.001.

The post-hoc Tukey’s test showed that number of correct reactions (NCR) during maximal workload of the incremental test (NCR_max_) decreased significantly (*p* < 0.05) by 9% in hypoxic conditions compared to normoxia. NCR_max_ values in hypoxia were significantly lower compared to NCR at rest (11.5%; *p* < 0.001) and NCR after 3 min of recovery after the incremental test (9.7%; *p* < 0.01) (Figure 4).

### 2.3. Brain-Derived Neurotrophic Factor and Selected Biochemical Variables

There was a significant interaction (condition x time of measure) effect in the BDNF (F = 3.66; *p* < 0.05) serum concentrations.

**Figure 5:**
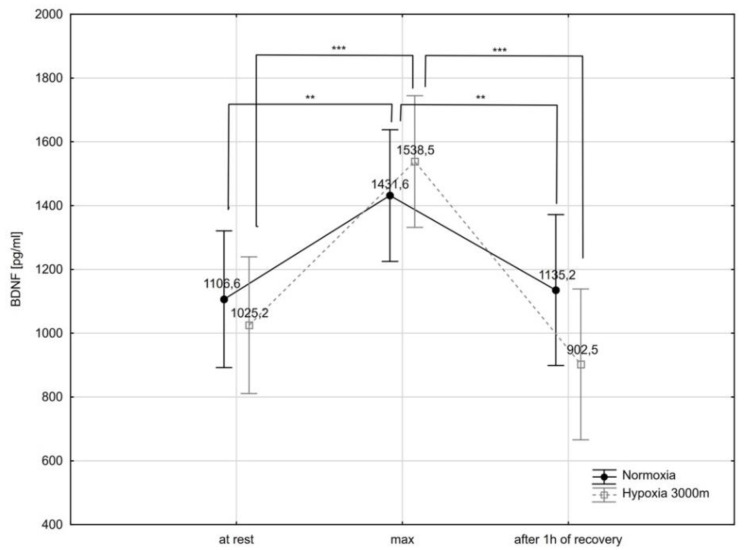
Brain-derived neurotrophic factor (BDNF) serum concentration at rest, during maximal effort (max) and after 1 h recovery of the recovery period in normoxia and hypoxia (3000 m). ** *p* < 0.01; *** *p* < 0.001.

The post-hoc Tukey’s test showed that BDNF concentration increased significantly immediately after the incremental test (BDNF_max_) in normoxia (by 29.3%; *p* < 0.01), as well as in hypoxia (by 50.0% *p* < 0.001). Additionally, BDNF concentration decreased significantly after a 1 h recovery period (BDNF_rest_) by 20.7% (*p* < 0.01) in normoxia and by 41.3% (*p* < 0.001) in hypoxia (Figure 5). There were no statistically significant differences in BDNF concentration between normoxia and hypoxia trials.

The Friedman test showed a statistically significant effect of the time of measurement on selected biochemical variables such as NO_2_^−^, C, adrenalin (A) and dopamine (DA) in both conditions (normoxia and hypoxia) (Table 1).

The post-hoc Friedman test showed that NO_2_^−^ concentration measured immediately after the incremental test was significantly higher (*p* < 0.05) compared to the NO_2_^−^ concentration observed at rest and 1 h after the incremental test in normoxia as well as hypoxia. However, the Wilcoxon test showed that were no significant differences between normoxia and hypoxia in NO_2_^−^ concentrations (Table 1).

There was no significant effect of the time of measurement (at rest, max, 1 h after test) on C concentrations in both conditions. However, there was a significant effect of conditions (normoxia vs. hypoxia) on C concentration. The Wilcoxon test showed that C concentration measured immediately after the incremental test (C_max_) was significantly higher (*p* < 0.05) by 20.7% in normoxic conditions compared to hypoxia (Table 1).

Moreover, the post-hoc Friedman test showed that A concentration measured immediately after the incremental test (A_max_) was significantly higher (*p* < 0.05) compared to the A concentration observed at rest (A_rest_; by 656.1%) and 1 h after the incremental test (A_1h after_; by 215,1%) in normoxia. However, in hypoxia, A_max_ and A_1h after_ were significantly higher (*p* = 0.001) compared to A_rest_ respectively by 505.9% and 86.4%. The Wilcoxon test showed that A_rest_, A_max_ and A_1h after_ in hypoxia were significantly higher (*p* = 0.001) compared to these values in normoxia, respectively by 368.2%, 275% and 534.2% (Table 1).

The post-hoc Friedman test showed that DA concentration measured 1 h after the incremental test (DA_1h after_) significantly decreased (*p* < 0.05) by 26.9% compared to the DA concentration measured immediately after the incremental test (DA_max_) in normoxia. However, in hypoxia, DA_max_ and DA_1h after_ significantly increased (*p* < 0.05) compared to the DA concentration at rest, respectively by 97% and 104.5%. Additionally, the Wilcoxon test showed that DA_max_ and DA_1h after_ in hypoxia were significantly higher (*p* < 0.01) compared to these values in normoxia, respectively by 69.2% and 140.3% (Table 1).

## 3. Discussion

Physical training is planned to improve physical fitness and performance, which confers numerous positive effects on the whole body function [69]. A pivotal role in regulation of these changes is assigned to the brain, particularly the prefrontal cortex, which takes part in regulation of many executive functions to prepare humans for situations demanding high levels of working memory, attention and cognitive flexibility [70]. On the other hand, altitude training, which nowadays has become a standard training protocol in many sports to increase exercise capacity [71,72,73], causes cognitive frailty [74]. The cognitive decline is most intensely manifested after acute hypoxic exposure and is more profound in athletes than non-trained individuals [75]. Therefore, the primary purpose of this study was to examine the impact of a single bout of exercise to volitional exhaustion during acute exposure well endurance-trained athletes to moderate hypoxia on psychomotor performance. We also measured serum BDNF, some selected hormones (C and catecholamines), the NO pathway metabolites (nitrite), as well as ET-1, as a possible candidate that may be involved in modulation of this phenomenon. We used measurements of CRT and NCR as indicators of psychomotor skills because these variables were demonstrated to be a dependable measure of cognition in different experimental approaches [22].

Results from this investigation revealed that exercise to volitional exhaustion extended CRT in both experimental conditions but only the impact of hypoxic conditions on this variable was statistically significant. A similar alteration, but with statistically significant changes in both cases, was seen in NCR (Figure 4). Additionally, NCR was significantly increased in response to exercise to volitional exhaustion performed in hypoxia as compared with normoxia. These results are in agreement with previous data reported by others which showed cognitive impairment of trained subjects at high altitude [75] and additionally suggested that NCR which specified response accuracy was a more sensitive tool for estimating cognitive state than CRT. Interestingly, the aforementioned exercise-induced effect was transient in both experimental conditions and studied variables returned to basal values within a few minutes after cessation of exercise.

Increasingly more evidence supports the action of BDNF as an underlying factor that elicits exercise/training-induced beneficial changes CNS [76,77,78]. Moreover, there are also suggestions that the negative impact on cognitive function in hypoxia can be at least partially explained by the simultaneous decrease of BDNF [79,80]. Studies with animal models have shown that this neurotrophin is produced, among other tissues, in brain by motor neurons [81] and intermittent hypoxia increases BDNF levels in neurons of the primary motor cortex [82]. Also, central BDNF cannot be measured in living humans. It has been suggested that the brain is the main source of the increased BDNF in circulation [83,84]. If so, and considering the fact that in our study similar circulating levels of BDNF were seen in both experimental groups but cognitive performance was suppressed in hypoxia, one could conclude that acute elevation of BDNF did not compensate for hypoxia-induced cognition impairment. Previous evidence suggests that BDNF plays a key role in memory and learning [85] and is a vital regulator of neuronal function and plasticity [86,87]. However, these aforementioned effects appeared almost selectively in response to a repetitive or chronic stimulus or in studies in vitro. Results of the current research are in line with reports of some studies using cognitive tasks such as executive function or attention which were unrelated to changes in BDNF after acute exercise [88,89,90]. One reason which at least theoretically might be considered for the acute action of BDNF is its involvement in synaptic transmission [91]. The results from animal studies indicate that during hypoxia neurons can within minutes alter synaptic transmission [92,93]. Support for a link between exposure to hypoxia and run-down of synaptic transmission has been widely documented in in vitro studies [94,95] and in rats subjected to severe (6100 m) hypoxia [96]. The mechanism by which severe hypoxia induced cognitive impairment was accompanied by a decrease in Acetylcholine (Ach) level and increase in Acetylcholinesterase (AChE) in the cortex [96].

Apart from BDNF, C has been identified as a possible endocrinological mediator of exercise which may modulate brain function [97,98]. Both hypoxia and exercise stimulate the gland cortex to release C. While it is well recognized that elevation of C has been observed in response to acute exercise of higher than moderate intensities, data from hypoxic studies are less consistent. In response to this environmental stimulus, increases or lack of changes have been reported. In our study, when maximal effort was performed in moderate hypoxia the C level was significantly lower compared to normoxia. As this hormone impairs the prefrontal cortex [99,100], the region of the brain that controls more of our cognitive function, one can argue that its deteriorative impact on psychomotor performance, if any, was negligible. However, there is a study showing similar exercised increased plasma C in normoxia and acute hypobaric hypoxia (3000 m altitude) [101]. This discrepancy may be the result of different research designs between aforementioned and our studies (normobaric vs. hypobaric hypoxia as well as cyclists vs. cross-country skiers and ice hockey players).

Previous studies demonstrated that physically active persons have shorter CRT than sedentary ones [102] and the regular U-shaped curve was obtained in athletes when CRT values were plotted against A and noradrenaline (NA) during graded incremental exercise to volitional exhaustion [34,62]. The latter results indicate that CRT exceeds the resting values at exercise loads close to maximal, and catecholamines may play a role in this phenomenon. Although most studies agree that catecholamine levels increase at rest and exercise at high altitude, brief or moderate hypoxia does not always elevate their levels [103,104], and this is especially true for NA [105]. The present finding reflects this phenomenon in the case of NA and serotonin (5-HT) while indicating elevated A as a potential player in cognitive control in our experimental paradigm. In favor of such an assumption is the significantly elevated A level during maximal effort in hypoxia with simultaneous statistically significant extension of CRT. Previous studies on patients with psychological trauma indicate that A and DA are involved in the activation of the prefrontal cortex [106] and similarly stress and aggressive behavior were shown to increase turnover of both A and DA in this area in rodents [107]. Acute hypoxia is considered as a systemic stress factor and was also seen to reduce cognitive function in rats, which was associated with DA signaling in the prefrontal cortex [63]. This finding is additionally supported by increased errors in the cognitive test, which were associated with reduced DA signaling in the prefrontal cortex [108]. However, the lack of significant differences in DA between our investigated groups probably excludes DA participation in the modulation of cognitive function in our subjects. It is worth noting that there is also a study [66] showing that during a short episode of anoxia, an increase in A level can have a protective effect against its disruptive effects. In the present study, this effect did not occur in response to exercise either in normoxia or in moderate hypoxia despite an elevated level of A in both cases. Collectively, the aforementioned results lead to the conclusion that A probably reveals biphasic action on cognitive function in hypoxic conditions, i.e., a positive effect during short-term and negative after prolonged actions.

It is well established that hypoxia releases a diffusible vasoconstrictor and vasodilator substance and this process can affect blood flow to the brain. Some evidence supports the view that an increase of blood flow to the brain may induce cognitive improvement by delivering elevated oxygenated hemoglobin [109]. In accordance with the above knowledge, we have measured in this research ET-1 and NO metabolites as the most potent representatives of endothelial released vasoconstrictors [110] and vasodilators [111], respectively. In humans, no changes in ET-1 response to maximal exercise in acute hypobaric hypoxia (3000) were noted as compared to normoxia [101]. In our study, both exercise and exercise in hypoxia also provoked no changes in ET-1, confirming occurrence of the above-mentioned effect in normobaric hypoxia, and suggesting that ET-1 had no important influence on blood low regulation in the acute response to moderate hypoxia.

Nitric oxide is mainly generated in the body by endothelial cells, but it is also produced in the CNS, where it is closely involved in neurotransmission and modulation of neuron metabolism [112]. A previous study conducted with diabetic patients indicated that increased bioavailability of NO was a factor that might enhance cognitive function [113]. However, the latest data questioned this possibility when psychomotor performance was tested in hypoxic conditions [114]. Our research conducted in a normobaric hypoxic chamber revealed an inconsiderable rise in serum NO_2_^−^ levels both in basal and exercise conditions as compared to normoxia. Since simultaneously cognitive performance was blunted it implied that NO production under these circumstances was likely too low to affect cognitive functions or NO was not a crucial player in this phenomenon. These assumptions are partially in line with recent findings which do not support a beneficial effect of NO_3_^−^ supplementation on cognitive function in sedentary males at moderate and very high simulated altitude [115]. However, it is clear that if during a profound reduction of absolute work under hypoxia the NO_2_^−^ level was higher than in normoxia then an additional exercise-independent system was responsible for NO_2_^−^ formation. This pathway is activated in parallel during exercise under hypoxic conditions, yet it needs to be identified.

Physical exercise involves markedly increased activity of many brain structures [116]. The metabolites produced in the muscles, which can diffuse into the CNS and can be utilized as a fuel to sustain increased energy requirements, may participate in this process [117]. Such possibility underscores the importance of the muscle produced lactate (LA) during exercise which can be transferred to neurons via monocarboxylate carriers and used in addition to LA delivered via astrocytes-neurons lactate shuttle [118] as energy fuel during neuronal activation as well. On the other hand, LA was recognized as a signaling molecule in the brain [119]. Among others, it can bind a receptor of the G protein coupled receptor family (GPRs) [120] and thereby cause a decrease of cyclic adenosine monophosphate (cAMP) level. This raises the possibility of interaction between LA and A in metabolism regulation on a subcellular level. If true, this phenomenon should have been more strongly connected with hypoxic conditions because A level during maximal effort in hypoxia was significantly elevated while lowering LA (as compared to controls). This was accompanied with simultaneous statistically significant extension of CRT.

## 4. Materials and Methods

### 4.1. Participants

Eleven cyclists (20 ± 1.4 years of age) were recruited for the study as volunteers. All participants had current valid medical examinations and showed no contraindications that would exclude them from the study. They declared that for at least one month before testing they did not take either medications or dietary supplements. Written informed consent was obtained prior to study commencement.

The basic anthropometric data of the volunteers (body height—BH, body mass—BM, fat content—FAT) are presented in Table 2. The experimental procedures involved, and the related risks were explained to all the participants verbally, informed written consent was taken from each participant and they could withdraw at any time of the study. The research project was conducted according to the Helsinki Declaration and was approved (no. 5/2013, approval date: 26.06.2013) by the Ethics Committee for Scientific Research at the Jerzy Kukuczka Academy of Physical Education in Katowice, Poland.

The subjects participating in the study were tested on two randomized occasions separated by 5 days duration in normoxic and hypoxic conditions. Participants were allocated to conditions using a computer-generated randomized list [121].

Hypoxic conditions were created using a normobaric hypoxia chamber (LOSA HYP/HYOP-2/3NU system, LOWOXYGEN SYSTEMS, Berlin, Germany) that is in use in the Laboratory of Hypoxia of the Jerzy Kukuczka Academy of Physical Education and the selected hypoxia was an equivalent of 3000 m altitude (FiO_2_ = 14.7%).

On each occasion, the participants were subject to two graded ergocycle tests each performed under normobaric normoxic and normobaric hypoxic conditions (3000 m asl). Before each test, body mass and body composition of each participant was determined using a model Inbody 720 (Biospace Co., Tokio, Japan) body composition analyzer using electrical impedance measurements.

### 4.2. Ergocycle Graded Exercise Test

The ergocycle tests were performed on a model Excalibur Sport (Lode BV, Groningen Netherlands) cycloergometer, beginning at a work load of 40 W, which was increased by 40 W every 3 min until volitional exhaustion. If a subject terminated the test before completing a given workload, then the maximum workload was calculated from the formula WRmax = WRk + (t/T × WRp) [122], where WRk—previous workload, t—exercise duration with the work-load until premature failure, T—duration of each workload, WRp—the amount of workload by which exercise intensity increased during the test.

During the tests, heart rate (HR), minute ventilation (VE), breathing frequency (BF), oxygen uptake (VO_2_) and carbon dioxide content in expired air (VCO_2_) were recorded in the subjects with the MetaMax 3B gas analyzer (Cortex, Leipzig, Germany). Fingertip capillary blood samples for the assessment of LA concentration (Biosen C-line Clinic, EKF-diagnostic GmbH, Barleben, Germany) were drawn at rest and at the end of each step of the test, as well as during the 3rd, 6th, 9th, and 12th minute of recovery. Additionally, capillary rest and post-exercise blood samples were used to determine acid-base equilibrium and oxygen saturation of hemoglobin (Rapid Lab 248, Siemens/Bayer Diagnostics, Erlangen, Germany).

### 4.3. Psychomotor Performance Determination

The choice reaction time and NCR were selected as indices of psychomotor performance as described previously [26]. Briefly, the CRT console was mounted on the wall in front of the ergometer at eye level, 1.5 m away from the subject. The test included 15 positive (red light or a sound) and 15 negative (green and yellow lights) stimuli applied in a randomized order in 1 to 4 s intervals. The subjects were asked to press and then to release, as quickly as possible, the button of the switch devised kept in the right hand in response to the red light, the button in the left hand in response to the sound and not react to the negative stimuli. The total time for each CRT was 107 s. The stimuli and the subjects’ responses were recorded using the reaction time measuring device (MRK 432, ZEAM, Zabrze, Poland). The reaction time was determined to the nearest 0.01 s. The results are presented at the mean reaction time of 15 responses to positive stimuli. The subjects were familiarized with the procedure a week before the study by practicing the task both at rest and during cycling.

### 4.4. Venous Blood

The participants were cannulated into the antecubital vein on the day of ergocycle testing 15 min prior to the breakfast. Venous blood samples (2 samples per time point) were collected 10 min later, then immediately after cessation of each ergocycle test, and 1 h after each ergocycle test. One sample of each pair was taken using ethylenediaminetetraacetic (EDTA) tubes (for morphology analysis); the other one was drawn using no anti-coagulant tubes and processed for serum for the other biochemical assays (BDNF, ET-1, C, catecholamines). After 30 min, blood samples were centrifuged at 1500× *g* for 15 min. The sera obtained were stored at −80 °C until analyzed.

### 4.5. Determination of Brain-Derived Neurotrophic Factor, Cortisol and Endothelin-1 Concentrations

Serum BDNF, C, and ET-1 concentrations were determined using a commercially available Quantikine ELISA kit (R&D Systems, Minneapolis, MN, USA) according to the procedure supplied by the manufacturer. This method allows measurement of BDNF, C, and ET-1 in the range of 0.372–4000 pg/mL, 0.030–100 ng/mL, and 0.031–50 ng/mL respectively. The intra-assay coefficient of variance was <4.0%, <8%, <4%, respectively. To quantify the level, a standard curve was performed using a standard solution.

### 4.6. Determination of Catecholamines by HPLC Method

Adrenalin, NA, DA and 5-HT were assayed in the serum using high performance liquid chromatography (HPLC, Gynkotek, Copenhagen, Denmark) with electrochemical detection using Coulochem III model 520 (ESSA, Copenhagen, Denmark).

Serum samples were mixed with 0.1 M perchloric acid containing 22.5 ng/mL ascorbic acid (ASC, Sigma-Aldrich, St. Louis, MO, USA). After centrifugation at 15,000 g, 10 min, at 4 °C, supernatant was filtered through a nylon syringe filter (Millipore, 0.22 μm, Merck KGaA, Darmstadt, Germany). Samples of 20 μL filtrate were injected into a high performance liquid chromatography system (Gynkotek, Copenhagen, Denmark) equipped with a Hypersil Gold (15 cm × 4.6 mm) column (Thermo Electron Corporation, Kleinostheim, Germany). The samples were eluted by a mobile phase made of 107 mM of Na_2_HPO_4_ × 2H_2_O, 107 mM citric acid, 0.3 mM octane-1 sulfonic acid sodium salt (OSA), 0.2 μM of EDTA, pH, 4.6, 1.5% methanol and 1.5% acetonitrile at a flow rate of 0.8 mL/min. The column temperature was set at 25 °C. Peaks were detected by electrochemical detection (Coulochem III, ESSA, Copenhagen, Denmark) at potentials of E1 = −50 mV and E2 = +400 mV. Data were collected and analyzed using Chromeleon software run on a PC (Gynkotek, Copenhagen, Denmark). DA and 5-HT contents in the sample were calculated by extrapolating the peak area from a standard cure.

### 4.7. Determination of Nitrite Concentration

Serum nitrite concentration were determined using a commercially colorimetric kit (R&D Systems, Minneapolis, MN, USA) according to the procedure supplied by the manufacturer.

### 4.8. Statistical Analysis

The results of the study were analyzed using Statistica 13.0 software (StatSoft, Cracow, Poland). The results are presented as arithmetic means (*x*) and standard deviations (SD). The statistical significance was set at *p* < 0.05. Prior to all statistical analyses, the normality of the distribution of variables was verified using the Shapiro-Wilk test. The paired sample t-test was used to determine the significance of differences in VO_2max_, WR_max_ between the two trials in different conditions. Furthermore, due to the lack of normality of distribution, the Wilcoxon test was used to determine the significance of differences in delta values in lactate concentrations (Delta LA) between the trials. The intergroup differences between the research trials (condition × time of measurement) were determined using the two-way ANOVA for repeated measures. When significant differences were found, the post hoc Tukey’s test was used. If the normality assumption was violated, the Friedman test was applied, whereas when significant differences were found, we used the post hoc Friedman test. The comparisons of repeated measurements (normoxia vs. hypoxia) were assessed by the Wilcoxon signed-rank test.

## 5. Conclusions

In conclusion, the results of this study showed that maximal physical exercise, regardless of whether it is performed under normoxia or mild normobaric hypoxia (equivalent of 3000 m altitude) caused a similar increase in BDNF concentration in the blood of well-trained athletes. Despite the fact that BDNF has been known to possess a protective effect on the brain, an elevated BDNF level did not protect our participants from cognitive impairment due to acute exposure to hypoxia, because indices of this variable, i.e., CRT and NCR, were worse in hypoxic conditions. All examined potential circulating factors that are known to affect BDNF expression/production (C, nitrite, ET-1, catecholamines, LA) most likely did not affect psychomotor functions in our experimental paradigm.

## Figures and Tables

**Table 1:** Mean values of selected biochemical variables registered in the different conditions (normoxia, hypoxia 3000 m) at rest, after incremental test (max) and after 1 h recovery period (after 1 h).

Variables	Normoxia (N)						Hypoxia 3000 m (H3)						Significance of Differences (* *p* < 0.05; ** *p* < 0.01)
	at Rest (1)		Max (2)		after 1 h (3)		at Rest (4)		Max (5)		after 1 h (6)		
	*x* ± SD	Me	*x* ± SD	Me	*x* ± SD	Me	*x* ± SD	Me	*x* ± SD	Me	*x* ± SD	Me	
**ET-1** (pg/mL)	2.5 ± 0.8	2.5	2.4 ± 1.7	2.3	2.0 ± 1.1	1.8	2.3 ± 0.8	2.5	2.3 ± 1.0	2.61	2.7 ± 1.2	2.5	
**NO_2_^−^** (pg/mL)	28.7 ± 12.1	23.2	47.6 ± 21.2	38.8	30.2 ± 12.7	25.6	33.8 ± 16.3	39.1	52.3 ± 16.3	49.8	29.5 ± 9.3	26.3	**N:** X^2^ = 13.81; *p* = 0.001 1–2 *; 2–3 * H3: X^2^ = 16.9; *p* = 0.002 4–5 *; 5–6 *
**C** (pg/mL)	8.7 ± 4.1	7.1	11.5 ± 3.8	11.1	12.4 ± 1041	9.8	7.2 ± 2.7	6.7	8.2 ± 2.48	8.8	9.4 ± 5.9	7.2	**N vs. H3:** 2–5 * (*U* = 29.0; *p* = 0.041)
**NA** (pg/L)	338.9 ± 213.4	337.2	555.6 ± 25.6	575.6	406.8 ± 28.4	329.3	334.6 ± 153.2	238.7	1109.2 ± 1045.4	348.5	575.2 ± 359	435.2	
**A** (pg/L)	53.9 ± 29.4	48.8	479 ± 358.1	369	112.5 ± 102.3	117.1	206.9 ± 70.7	228.5	1436.1 ± 622.9	1384.7	827.4 ± 263.7	742.7	**N:** X^2^ = 16.9; *p* = 0.002 1–2 *; 2–3 * H3: X^2^ = 18.2; *p* = 0.001 4–5 *; 4–6 * N vs. H3: 1–4 ** (*U* = 1.0; *p* = 0.001) 2–5 ** (*U* = 9.0; *p* = 0.001) 3–6 ** (*U* = 0.0; *p* = 0.001)
**DA** (pg/L)	7.6 ± 10.9	4.3	8.9 ± 5.8	7.8	5.6 ± 1.4	5.7	7.3 ± 2.5	6.7	12.5 ± 2.7	13.2	12.1 ± 5.5	13.7	**N:** X^2^ = 10.1; *p* = 0.006 2–3 * H3: X^2^ = 10.4; *p* = 0.005 4–5 *; 4–6 * N vs. H3: 2–5 ** (*U* = 17.0; *p* = 0.008) 3–6 ** (*U* = 16.0; *p* = 0.006)
**5-HT** (pg/L)	155.1 ± 104.2	135.4	212.6 ± 148.1	150.3	192.1 ± 90.1	190.7	136.5 ± 67.1	156.7	205.9 ± 124.3	150.4	156.4 ± 82.1	148.3	

*x*—arithmetic means; SD—standard deviations; Me—median; ET-1—endothelin-1; C—cortisol; NA—noradrenaline; A—adrenalin; DA—dopamine; 5-HT—serotonin; * *p* < 0.05; ** *p* < 0.001.

**Table 2:** Mean values of body height (BH), body mass (BM) and fat content (FAT) of study participants (*n* = 11).

BH (cm)	BM (kg)	FAT (%)
180.5 ± 6.5	70.3 ± 6.8	9.4 ± 3.1

## Abbreviations

BDNF	brain-derived neurotrophic factor
ET-1	endothelin-1
5-HT	serotonin
CRT	choice reaction time
NCR	number of correct reactions
DA	dopamine
LA	lactate
NA	noradrenaline
C	cortisol
A	adrenalin
